# Providing health care in conflict settings: a call for papers

**DOI:** 10.2471/BLT.19.232769

**Published:** 2019-05-01

**Authors:** Flavio Salio, Altaf Musani

**Affiliations:** a World Health Organization, Yemen Country Office, Algiers Street, P.O. Box 543, Sana’a, Yemen.

People living in conflict-affected areas incur physical and psychological trauma. They are also vulnerable to disease outbreaks and disruptions in the supply of food and water, medicines and health services. High-quality, accessible medical care is very difficult to provide in such settings. The Geneva Conventions hold warring parties accountable for the provision of care to civilians and combatants. However, these parties may not be willing or able to provide medical assistance to conflict-affected populations. Recent major conflicts, such as in Mosul, Iraq and Al Raqqa, Syrian Arab Republic, have moved health and humanitarian assistance closer to the frontline and challenged the risk-averse approach of traditional responders.

Accountability to affected populations, as defined by the Inter-Agency Standing Committee,^1^ requires humanitarian responders to provide quality assistance in a timely manner while upholding best practices. Medical ethics and international humanitarian law also bind health practitioners to an operational and accountability framework. When warring parties fail to provide care and assistance to civilians, there is still an imperative to save lives and reduce suffering.

The recent experiences of humanitarian responders show that operational guidance on the provision of trauma care in conflict settings is needed. These experiences also constitute valuable lessons, from which best practices can be identified and future responses improved. An assessment of response in Mosul found that the “WHO-coordinated efforts helped address critical needs in the provision of trauma care for wounded civilians and saved lives.”^2^^,^^3^ However, the referral pathway used in this response created a complex system of care that could not be used by all humanitarian actors because of proximity to the frontlines and to military personnel.^2^ WHO recognized the operational, technical and ethical dilemmas of the response, trying to balance battlefield care and medical ethics with the humanitarian principles of neutrality, impartiality, humanity and independence.^4^ WHO emphasized its role as a provider of last resort, and called for partners to work closer to the frontlines. The debate generated by the referral pathway shows that research is needed on issues of quality of, and access to, timely trauma care, on prevention of attacks on health-care workers, transport, patients and facilities, as well as on outbreak prevention and response.

Most research on trauma care in conflict settings has been done in the context of symmetric warfare, when humanitarian agencies have equal access to all warring parties and the wounded, and where the military is the main provider of care. This research led to changes in care provision at the frontline, where the risk of functional impairment is usually highest. Rapid evacuation from the location of injury to the care facility is needed to save lives and reduce disabilities. Despite hard-won experience gathered by many military, United Nations, nongovernmental, civilian and humanitarian actors, there is very limited research on health-care provision and civilian protection in asymmetric warfare, where humanitarian agencies have no access to one or more warring party.

To build the evidence base for future responses, the *Bulletin of the World Health Organization* encourages submissions to its research, policy-and-practice and lessons-from-the-field sections to shape policy and improve health outcomes in conflict settings.
